# Clinical management of psychotic pregnancy denial: what do we know? Case report and narrative review.

**DOI:** 10.1192/j.eurpsy.2023.2400

**Published:** 2023-07-19

**Authors:** M. Martín Velasco, I. Romero Gerechter

**Affiliations:** Psychiatry, Hospital Universitario Príncipe de Asturias, Alcalá de Henares, Spain

## Abstract

**Introduction:**

Denial of pregnancy is the lack of awareness of being pregnant. It associates with increased morbidity and mortality of mother and child and can be classified as non-psychotic or psychotic. There is few literature regarding the latter, making it difficult to recognize, let alone to treat, since we do not have robust data on the incidence nor approved interventions.

**Objectives:**

To get a better understanding on the standard of care for patients with psychotic denial of pregnancy.

**Methods:**

We present a case report alongside a narrative literature review on the topic.

**Results:**

We report the case of a 39-year-old caucasian woman, foreign, undomiciled, who was admitted to a Psychiatry unit due to psychotic symptoms. Her birthplace and prior medical records were unknown. She did not recognize being pregnant and showed great irritability when asked; her responses ranged from delusional attributions of symptoms related to the pregnancy to partially acknowledging her state but refusing to answer questions. Obstetric ultrasound revealed a low risk 35 weeks pregnancy. Treatment included quetiapine up to 700mg daily and psychological approach. A multidisciplinary team managed the case and arranged a plan for delivery. Eventually, delusional symptoms remitted and she accepted the gestation. She showed full collaboration during delivery, giving birth to a healthy female and presented transient recovery. After being separated from her daughter, her clinical situation worsened.

Psychotic denial of pregnancy is rather uncommon. It is usually seen in patients with prior history of major mental illness, most frequently schizophrenia, and important psychosocial vulnerability. It associates with several negative outcomes for mother and baby, including neonaticide. Most studies agree on the need of a multidisciplinary intervention including obstetrics, psychiatry, and others (social agents, ethical consultants…) to generate a plan for mother and baby. Biopsychosocial aspects should always be considered and each case individually formulated. Pregnant women must be given clear and concise information about the process. For some, seeing obstetric ultrasound might help them accept the pregnancy. Some authors propose labour induction prior to 39 weeks and performing a C-section, especially in cases of uncontrolled psychosis or risk of noncompliance. Most studies also recommend antipsychotic treatment. In cases of persistent denial or acute crisis, especially during the third trimester, patients should be admitted to a psychiatry unit with easy access to obstetric care. Supportive psychotherapy and psychosocial intervention should try to identify precipitating stressors for denial, such as prior or anticipated custody loss, which has been linked to psychotic denial.

**Conclusions:**

Psychotic denial is a serious illness which requires a multidisciplinary treatment including biopsychosocial and obstetrical aspects.

**Disclosure of Interest:**

None Declared

